# Does the Width of the Bony Cochlear Nerve Canal Predict the Outcomes of Cochlear Implantation?

**DOI:** 10.1155/2018/5675848

**Published:** 2018-03-21

**Authors:** Juyong Chung, Jeong Hun Jang, Sun O Chang, Jae-Jin Song, Sung-Woo Cho, So Young Kim, Jun Ho Lee, Seung-Ha Oh

**Affiliations:** ^1^Department of Otorhinolaryngology-Head and Neck Surgery, Wonkwang University College of Medicine, Iksan, Republic of Korea; ^2^Department of Otolaryngology, Ajou University School of Medicine, Suwon, Republic of Korea; ^3^Department of Otolaryngology-Head and Neck Surgery, Kangbuk Samsung Hospital, Sungkyunkwan University School of Medicine, Seoul, Republic of Korea; ^4^Department of Otorhinolaryngology, Seoul National University Bundang Hospital, Seoul National University College of Medicine, Seongnam, Republic of Korea; ^5^Department of Otorhinolaryngology, Seoul National University College of Medicine, Seoul, Republic of Korea; ^6^Department of Otorhinolaryngology, CHA Bundang Medical Center, CHA University, Seongnam, Republic of Korea; ^7^Sensory Organ Research Institute, Seoul National University Medical Research Center, Seoul, Republic of Korea

## Abstract

A narrow bony cochlear nerve canal (BCNC) is associated with sensorineural hearing loss necessitating cochlear implantation (CI). This study evaluated the implications of BCNC width for post-CI outcomes. A total of 56 children who had received CIs were included. The patients were divided into three groups according to the width of the BCNC (Group 1: diameter < 1.4 mm, *n* = 17; Group 2: diameter 1.4–2.0 mm, *n* = 14; Group 3: diameter > 2.0 mm, *n* = 25). The post-CI speech performances were compared among the three groups according to BCNC width. The correlation between BCNC width and post-CI speech performance was evaluated. Logistic regression analysis was also performed to investigate factors that can impact post-CI speech performance. Cochlear nerve deficiency (CND) occurred more frequently in Group 1. Groups 1 and 2 had significantly worse post-CI outcomes. Patients with intact cochlear nerves had significantly better post-CI outcomes than those with CND. When the cochlear nerve was intact, patients with a narrower BCNC showed less favorable results. Therefore, patients with either a narrow BCNC or CND seemed to have poorer outcomes. A narrow BCNC is associated with higher CND rates and poor outcomes. Measurement of BCNC diameter may help predict CI outcomes.

## 1. Introduction

The cochlear implant (CI) is an innovative device that is used to treat patients with bilateral, severe, or profound sensorineural hearing loss. It converts the auditory signal into an electrical signal, which in turn stimulates spiral ganglion neurons (SGNs), thus transmitting the signal to the auditory brainstem via the cochlear nerve. Therefore, the integrity of the cochlear nerve is the main factor affecting improvement in speech performance after CIs. This can be evaluated before surgery using magnetic resonance imaging (MRI) or the promontory stimulation test (PST).

**Figure 1:**
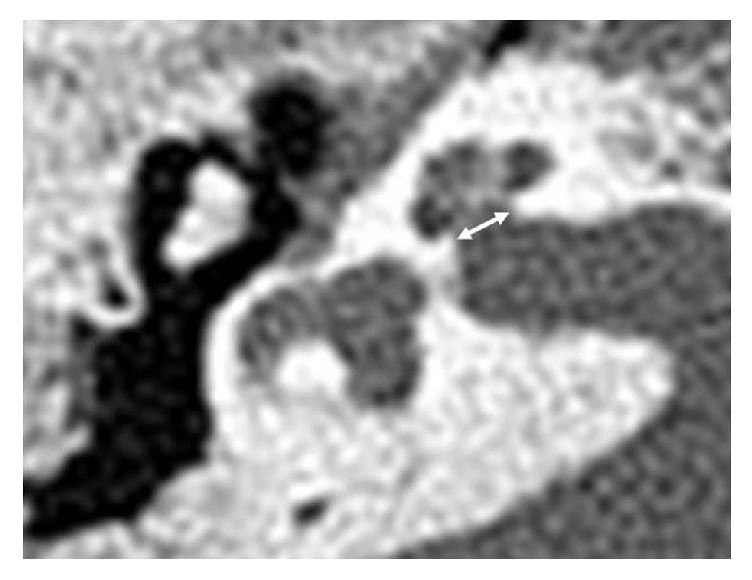
Axial slices of temporal bone computed tomography for the measurement of bony cochlear nerve canal (BCNC) in normal bony cochlear nerve canal. The width was measured by the distance between the inner margins of bony walls at midportion, at the fundus level of cochlear nerve in internal auditory canal.

The bony cochlear nerve canal (BCNC) lies between the fundus of the internal auditory canal (IAC) and the base of the cochlea. It encases the cochlear nerve fibers from the spiral ganglion to the cochlear nerve [1] (Figure 1). Therefore, a narrow BCNC likely indicates anatomic or functional deficiency in the cochlear nerve. Specifically, if the width of the BCNC—the distance between the inner margins of the bony walls at the midportion—is less than 1.4 mm, then the cochlear nerve may be abnormal [2]. One study found such a narrow BCNC in approximately 60% of patients with unilateral sensorineural hearing loss (SNHL) [3]. Furthermore, BCNC stenosis may be related to cochlear nerve hypoplasia [3–5]. It follows that patients with BCNC stenosis may not benefit from CI and that the condition may be a predictor of poor post-CI outcome [2]. However, the relationship between BCNC diameter and post-CI outcome has not been characterized in previous studies.

In the present study, we aimed to evaluate the width distribution of the BCNC in ears that had received CIs, to evaluate the association between BCNC width and cochlear nerve deficiency (CND), and to analyze the correlation between BCNC width and speech performance after CI. This information may be helpful in predicting improvements in speech performance after CI.

## 2. Materials and Methods

### 2.1. Subjects

This retrospective study was approved by the Institutional Review Board of the Clinical Research Institute (SNUH IRB number 1006-099-322). The need for informed consent was waived. A total of 452 children who had received a CI between January 2005 and April 2012 were enrolled. Their medical records were reviewed retrospectively for data regarding otorhinolaryngological examinations, evaluation of preoperative hearing status, pre- and postoperative speech performance, temporal bone computed tomography (TBCT), and magnetic resonance imaging (MRI) of the IAC. Among these 452 patients, children satisfying one of following criteria were excluded: (1) follow-up less than 3 years, (2) insufficient medical records, (3) either MRI or TBCT unavailable, (4) device failure or incomplete electrode insertion, and (5) children with inner ear anomaly, as classified by the system published by Sennaroglu et al. [6]. The CT findings of all ears included in this study were normal except for differences in the BCNC width. After all exclusions, our cohort was comprised of 56 children who were followed up for more than 3 years, had both available TBCT and MRI of the IAC, and had no inner ear anomaly except for differences in the BCNC width.

Ultimately, 56 children were classified into three groups according to the BCNC width. BCNC stenosis was defined as a BCNC width less than 1.4 mm, as reported in a previous study [2]. When the BCNC was greater than 2.0 mm in diameter, it was defined as having a normal size, as described in previous research [1]. Children in this study were divided into three groups based on the BCNC width: Group 1 (<1.4 mm, *n* = 17), Group 2 (1.4–2.0 mm, *n* = 14), and Group 3 (>2.0 mm, *n* = 25). The mean ages at the time of CI in the three groups were 27.92 ± 15.92 months, 29.32 ± 9.84 months, and 33.43 ± 15.7 months, respectively. The mean follow-up periods were 27.17 ± 13.66 months, 47.20 ± 16.69 months, and 49.64 ± 21.91 months, respectively.

### 2.2. Measurements

In this study, two parameters were used to analyze the correlation between the BCNC and the post-CI outcome: BCNC width and the status of cochlear nerve. The BCNC width was estimated using the axial plane of the TBCT. The axial plane runs parallel to the infraorbitomeatal line. As reported in a previous study, the width of the BCNC was measured at its midportion and was defined as the distance between the inner margins of the bony walls (Figure 1) [3, 7]. We evaluated the status of the cochlear nerve at the lateral part of the IAC using the MRI images. At this site, the facial and cochlear nerves are of similar size and larger than the vestibular nerve [8, 9]. Therefore, CND was defined when the cochlear nerve at the lateral aspect of IAC was smaller than (1) the superior or inferior vestibular nerve and (2) the facial nerve at the same point. All measurements were performed separately by two otologists—J. H. J., who had 9 years' experience, and J. C., who had 8 years' experience in otorhinolaryngology. They estimated the BCNC width and the status of the cochlear nerve using a computer-based caliper in the PACS system; they were blinded to the medical history of all the children. To evaluate post-CI speech performance, we reviewed (1) the preoperative categories of auditory performance (CAP) score, (2) the CAP score at 6, 12, 24, and 36 months postoperatively, (3) open-set test results (word/sentence), and (4) the Korean picture vocabulary test (K-PVT) percentage. The K-PVT is similar to the Peabody PVT-revised edition; it evaluates the receptive vocabulary of Korean children, with reference to their age and to the population with normal hearing. The examiner speaks one word that describes one of four pictures and asks the individual to say the number of picture or point to it. The result is expressed as a percentile score, which is then compared with the scores of children of the same age who have normal hearing.

### 2.3. Data Analysis

The post-CI speech performances were compared among the three groups using the Mann–Whitney–Wilcoxon test. The correlation between BCNC width and post-CI speech performance was evaluated using Pearson correlation analysis. The association between the BCNC width and speech performance was assessed using multiple logistic regression. All analyses was performed using SPSS version 17.0 (SPSS Inc., Chicago, Illinois), and the 95% confidence intervals were also assessed. In all analyses, *p* values < 0.05 were considered statistically significant.

## 3. Results

### 3.1. Factors Influencing the Outcomes of CI in the Three Groups

There were no significant differences among the groups in age at the time of CI, duration of hearing aid use, or residual hearing. The mean BCNC width in Group 1 was 0.91 ± 0.32 mm, that in Group 2 was 1.89 ± 0.17 mm, and that in Group 3 was 2.34 ± 0.14 mm. Most differences among the groups were not statistically significant; however Group 1 had a higher rate of CND (13/17; 76%) than Group 2 (3/14; 21%) and Group 3 (0/25; 0%; Table 1).

### 3.2. Post-CI Speech Performance according to BCNC Width

**Figure 2:**
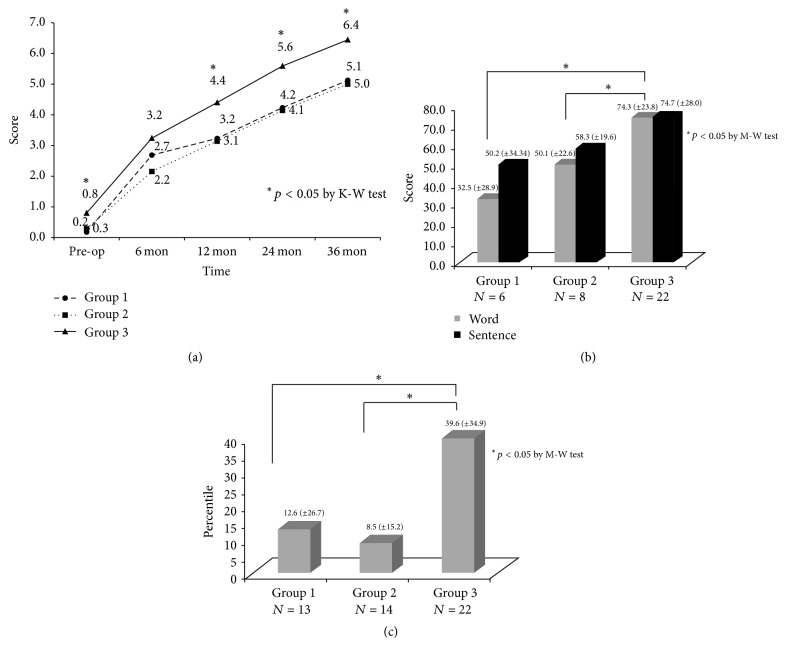
Comparisons of post-CI speech performance between three groups. *∗* means statistically significant. (a) CAP score. Since 12 months after CI, Group 3 showed significantly better outcomes than Groups 1 and 2. (b) Open-set score. Groups 1 and 2 showed less favorable results. (c) Picture vocabulary tests.

We compared the speech performance among the groups. In Groups 1 and 2, the CAP score 36 months after CI was lower than that in Group 3 (Figure 2(a)). With regard to the open-set score 24 months after CI, Groups 1 and 2 scored worse than Group 3 (Figure 2(b)). We saw a similar trend in the K-PVT 24 months after CI, in which Groups 1 and 2 continued to underperform (Figure 2(c)).

### 3.3. Postoperative Speech Performance—according to BCNC Width—of CI Patients with an Intact Cochlear Nerve

**Figure 3:**
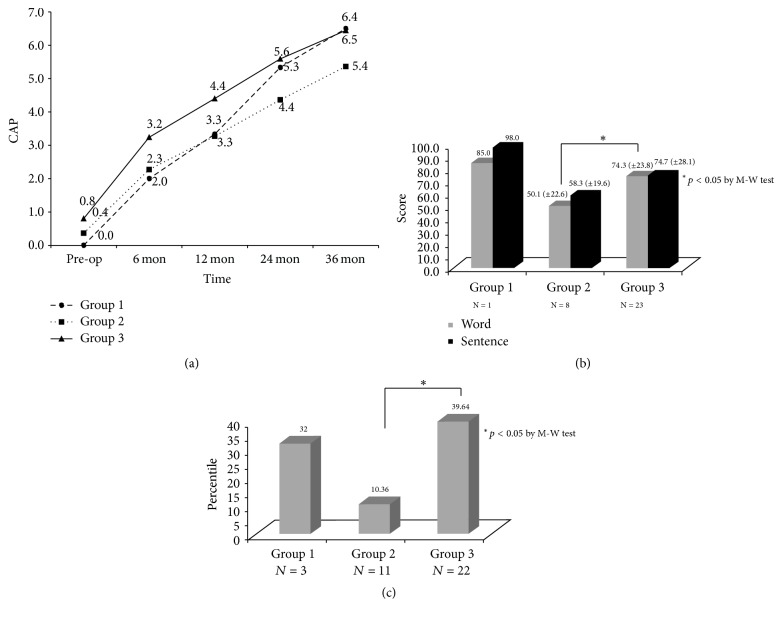
Comparisons of post-CI speech performance between three groups when the CN is present. *∗* means statistically significant. (a) CAP score. (b) Open-set score. (c) Picture vocabulary test.

To rule out confounding factors related to CND, we investigated the post-CI speech performance according to BCNC width when the cochlear nerve was present. To do so, we referred to the parasagittal constructive interference in the steady state MRI images of the IAC. There were no significant differences in CAP score between the groups. However, the open-set sentence score of Group 2 was lower than that of Group 3. Group 1 contained only one patient and therefore had few statistical power. The trend continued in the K-PVT (Figure 3).

### 3.4. Correlation between BCNC Width and Post-CI Speech Performance

**Figure 4:**
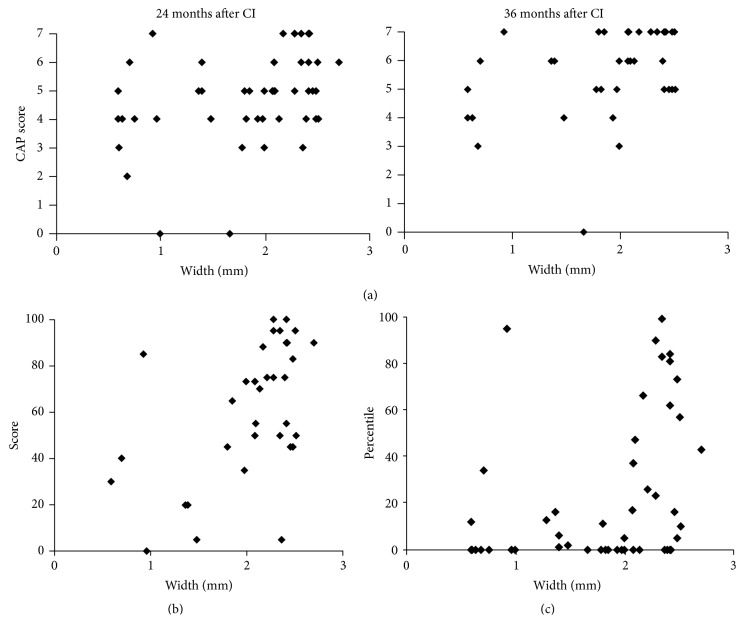
Correlation of width of BCNC with post-CI speech performances was shown. (a) The CAP score 24 and 36 months after CI was correlated linearly with BCNC width. (b) Positive correlation between the width of BCNC and open-set word score at 24 months after CI. (c) Positive correlation between width of BCNC and Picture vocabulary test at 24 months after CI.

We analyzed the implications of BCNC width for post-CI speech performance using Pearson correlation analysis. The Pearson correlation coefficients were calculated between BCNC width and various measures of post-CI speech performance such as CAP score, open-set word or sentence score, and K-PVT results. The CAP score 24 and 36 months after CI was correlated linearly with BCNC width (Figure 4(a)). In CAP score at 24 months and 36 months post-CI, the correlation coefficient of 0.377, 0.395 demonstrates a linear relationship between the two variables (*p* < 0.05). Furthermore, BCNC width and open-set word score 24 months after CI showed a positive correlation (correlation coefficient = 0.533, *p* < 0.05), and BCNC width was positively correlated with K-PVT 24 months after CI (correlation coefficient = 0.342, *p* < 0.05) (Figures 4(b) and 4(c)). These findings suggest that preoperative measurement of the BCNC width using CT images might be related to post-CI outcomes and that such measurement provides clinicians with useful information for counseling parents and patients.

### 3.5. Factors Determining the Auditory Performance after CI

We used multiple regression analysis to ascertain which factors influence post-CI speech performance in Group 1, Group 2, and Group 3. We analyzed five potential influencing factors: (1) age at the time of CI, (2) BCNC width, (3) presence of CND, (4) duration of hearing aids used before CI, and (5) residual hearing. The results of the analysis varied depending on the specific test used to evaluate speech performance; nonetheless, BCNC width was significantly associated with open-set sentence score after CI (Table 2). In addition, the presence of CND was statistically associated with K-PVT results, and the duration of hearing aids used before CI was significantly related to CAP score (Table 2). However, age at the time of CI and residual hearing were not statistically associated with CI outcomes.

## 4. Discussion

In a previous study, we evaluated the BCNC length and width in normal inner ears: 1.07 mm and 2.38 mm, respectively. In cases of congenital unilateral sensorineural hearing loss, the BCNC length and width in affected inner ears were significantly smaller than those in normal inner ears [1]. The mean width in the affected inner ears in our previous study was 1.58 mm; other investigators have reported mean widths of 1.12 mm and 1.0 mm [10, 11]. Many authors have reported that the BCNC length in normal hearing inner ears ranges from 0.93 mm to 1.17 mm and that the width ranges from 1.88 mm to 2.13 mm (Table 3) [2, 11, 14]. In 2000, Fatterpekar et al. [3] reported that the width of the BCNC was significantly smaller in patients with SNHL than in a control group. One year later, Nelson and Hinojosa [14] recounted histological evidence of cochlear nerve aplasia in the normal inner ear and IAC. In 2006, Adunka et al. [15] stated that cochlear nerve aplasia is not always associated with IAC hypoplasia and that both high-resolution MRI and CT must be performed in patients with profound SNHL. Komatsubara et al. [10] reported that cochlear nerve aplasia may be present when the canals are narrower than 1.5 mm on TBCT; such aplasia can definitively be seen using MRI. In all these efforts to ascertain the significance of BCNC width, clinicians took note of the clinical implications of BCNC width in CI candidates.

Previous research has suggested that the lower limit of normal BCNC width is 2.10 mm; this was obtained by subtraction of the standard deviation from the mean value in normal hearing inner ears [1]. Thus, the mean BCNC width in normal patients is higher than 2.0 mm. When we divided the patients of the present study into three groups based on the BCNC width, Group 3 (normal BCNC width) comprised all patients whose BCNC was wider than 2.0 mm.

Recently, there have been more requests for imaging to evaluate inner ear anomalies and predict post-CI outcomes. High-resolution CT (HRCT) of the temporal bone provides additional information regarding temporal bone pathology, facial nerve position, and inner ear malformations [4]. Furthermore, MRI is better able to show the labyrinth and eighth cranial nerve [4]. Thus, MRI is the best tool to evaluate the status of the cochlear nerve in cases of aplasia or hypoplasia and to predict negative outcomes in CI candidates [15, 16].

Imaging studies provide useful information regarding inner ear anomalies. Sennaroglu [6] classified inner ear malformations to investigate their etiology. However a classification of this kind may not be sufficient to predict CI outcomes, because it does not include IAC malformations, such as narrow IAC and BCNC hypoplasia, which have a negative impact on CI outcomes because they indicate CND [4, 17]. Recently, some authors have reported a relationship between BCNC stenosis and cochlear nerve hypoplasia [3, 10, 11, 18]. In fact, BCNC stenosis may be secondary to cochlear nerve hypoplasia [11]. Stjernholm and Muren [2] stipulated that a cochlear nerve abnormality was a possibility when the BCNC was less than 1.4 mm in diameter. In a report by Komatsubara et al. [10], patients with a narrow BCNC on CT were diagnosed as having cochlear nerve hypoplasia, which was confirmed using MRI, with 88.9% sensitivity and 88.9% specificity. The same authors stated that when the BCNC was less than 1.5 mm on CT, cochlear nerve hypoplasia could be seen on MRI. In a report by Kono [11], a BCNC diameter less than 1.7 mm suggested cochlear nerve hypoplasia, even when no cochlear abnormality could be found on CT. BCNC stenosis with a diameter of 1.5 mm or less suggests cochlear nerve hypoplasia or aplasia. On the other hand, cochlear nerve hypoplasia was not seen in children who had BCNC stenosis with a diameter greater than 1.5 mm. A previous study indicated that children who had BCNC stenosis with a diameter of 1.5 mm or less and those who had severe inner ear malformations on HRCT require MRI of the cochlear nerve [4]. Therefore, noting the presence of BCNC stenosis confirms a diagnosis of cochlear nerve hypoplasia or aplasia [18]. Moreover, BCNC stenosis with a diameter of 1.5 mm or less further confirms such a diagnosis [10].

In the present study, CND was frequently associated with a narrow BCNC, especially one less than 1.4 mm in diameter; therefore, measurements of the BCNC may help in predicting the outcomes of CI. In addition, if BCNC stenosis or cochlear malformation is revealed on HRCT, additional MRI may show cochlear nerve aplasia or hypoplasia. Thus, MRI can confirm the status of the cochlear nerve, whether aplastic or hypoplastic, and help predict improvements in hearing performance after CI. In turn, BCNC stenosis could be used to select children who should undergo further evaluation using MRI. These findings have important implications for clinicians who evaluate children with SNHL.

Several case reports have included information on the speech performance of patients with CND. Recent reports of CI among children with CND have been reported with generally poor results. Buchman et al. reported post-CI speech performance among 22 children with CND [19]. Children with CND had higher pure tone averages and required greater charge for CI stimulation than other inner ear malformation types [19]. In addition, open-set speech perception after CI was achieved in only 19% of CND cases and participating in mainstream education is more limited [19]. Zanetti et al. reported one case of CND after CI. In this report, although the child scored poorly in every perceptive category when using the CI alone, the device greatly enhanced his speech understanding when he also used a hearing aid in his opposite ear [20].

The present article is the first to report a correlation between BCNC width and post-CI speech performance. In addition, we performed multivariate logistic regression analysis to evaluate influencing factors. The speech performance after CI may be influenced by several factors [21]. The comprehensive evaluation of prognostic factors enables CI team to counsel the CI candidates for the post-CI outcome accurately. It was known that the physiologic factors (age at CI, duration of deafness, meningitis, genetic mutation), the anatomical factors (inner ear anomaly, BCNC, CND), functional factors (residual hearing level, preoperative use of hearing aid), device factors (coding strategy, brand of CI, the percentage of active electrode), and education/rehabilitation factors (mode of communication, socioeconomic status, post-CI rehabilitation service, family support) could be related to the CI outcome [22]. In this current study, BCNC, CND, and the duration of hearing aids use were related to post-CI outcome. On the other hand, the age at CI did not have an effect on speech performance after CI because most children were implanted at the age less than 2 years (age at CI < 2 years, 73%), which is consistent with previous studies [23, 24]. This study was mainly focused on the relationship between the BCNC and CI outcome, so several factors such as meningitis, genetic mutation, device factors, and education/rehabilitation factors were not analyzed.

Besides age at the time of CI and preoperative residual hearing, which were already known as prognostic factors for CI outcome, BCNC width was correlated with speech performance in a 3-year postoperative follow-up. The present study showed a positive correlation between post-CI long-term speech performance and BCNC width. These data suggest that BCNC width indirectly reflects the residual capability of the cochlear nerve. Hence, along with MRI of the IAC MRI, TBCT may contribute to preoperative evaluation of cochlear nerve residual capabilities, and it may be helpful in patient counseling.

The fundamental goal of preoperative imaging is the prediction of CI outcomes. In this regard, the results of the present study provide indirect evidence that TBCT is useful in CI candidates. Generally, MRI is recommended in the evaluation of cochlear nerve integrity in patients with profound SNHL; however, the estimation of BCNC using TBCT may play a supportive role. That is, narrow BCNC may indicate higher rates of CND and poor post-CI outcomes. Therefore, preoperative measurement of the BCNC on CT images may help to predict CI outcomes.

In the present study, we found that the frequency of CND was much higher in CI patients with BCNC hypoplasia (76%) than in CI patients with a normal BCNC (21%). Moreover, the width of the BCNC was significantly smaller in CI patients with CND (1.11 mm) than in CI patients with a normal cochlear nerve (2.08 mm). These correlations may be partially due to developmental abnormalities. We also tried to ascertain the relationship between BCNC width and post-CI speech performance. To understand the significance of BCNC width in this regard, we must understand the embryology of the inner ear and IAC. The exact cause of the narrow BCNC in patients with SNHL is not known. We assume that deficiencies in the development of the otic vesicle inhibit normal nerve growth factor production. This may in turn result in excessive neuronal degradation and prevent normal growth of the cochlear nerve. Furthermore, because most patients with profound SNHL are thought to have anomalies of the membranous labyrinth [1], this inner ear malformation may inhibit the normal trophic effects of nerve growth factor, causing a small cochlear nerve and hypoplasia of the BCNC. As the IAC develops—at 9 weeks' gestation—the mesenchyme surrounding the otic vesicle begins to chondrify, finally forming the otic capsule by means of ossification. Therefore, the IAC is formed through the inhibition of cartilage formation at the medial aspect of the otic vesicle; the vestibulocochlear nerve mediates this inhibition by inducing nerve growth factor. In the absence of the nerve, a canal will not be formed [24, 25]. However, Casselman et al. [8] found that cochlear nerve hypoplasia and aplasia can occur with or without labyrinth anomalies. Several other authors have reported cases of cochlear nerve aplasia with normal IAC dimensions [18, 26]. Therefore, the etiology of acquired CND may be complex.

CND can result from degeneration of the nerve fibers in the IAC after cochlear injury (vascular, traumatic, compressive, or inflammatory injury). In the case of CND with a normal BCNC, it may be that the vestibulocochlear nerve is injured in one of the ways mentioned—that is, long after BCNC formation. The destruction of the cochlear neuroepithelium may lead to retrograde destruction of the spiral ganglia in the modiolus [27]. On the other hand, when the cochlear nerve is injured microscopically during the late stages of IAC formation (approximately 5 months' gestation) or when it has abnormalities related to growth factors, hypoplasia of the BCNC may result, even in cases of relatively intact cochlear nerve development.

In the present retrospective review, we found that the frequency of BCNC hypoplasia in CI patients without inner ear anomalies was 3.8% (17/452) and that the BCNC width was correlated with long-term speech performance (more than 24 months) after CI. Furthermore, the frequency of CND was 3.5% (16/452), and the probability of CND diagnosis by MRI is significantly increased when BCNC hypoplasia is diagnosed by TBCT. Patients who had both BCNC hypoplasia and CND tended to have poor speech performance after CI, and CND was frequently associated with a narrow BCNC. Therefore, a narrow BCNC may indicate higher rates of CND and poor outcomes. According to a recent study, BCNC stenosis is significantly associated with impaired speech discrimination; this would be expected if BCNC abnormalities indicated cochlear nerve dysfunction. Therefore, BCNC stenosis predicts poor outcomes for auditory rehabilitation, and BCNC measurement may help to predict CI outcomes.

The cochlear nerve size is thought to be associated with the population of spiral ganglion cells. Therefore, determining the caliber of the nerve may be helpful in predicting the outcome of CI [28]. In one previous study, even when the cochlear nerve was thin, it still effectively transmitted impulses to allow hearing; therefore, MRI depiction of CND is considered a relative contraindication for CI [29]. However, because CT and MRI are limited, patients with CND in the present study were further assessed by electrical auditory brainstem response testing and behavioral audiometry to check for residual hearing. Patients with a response received a CI. Thus, electrophysiological testing, such as the PST or intracochlear, electrically evoked auditory brainstem response, might have prognostic value in predicting the outcome of CI in patients with a narrow BCNC. Therefore, clinicians need to determine the anatomical status of the cochlear nerve by evaluating the BCNC width using TBCT or MRI. They must also (1) accurately analyze the implications of preoperative electrophysiological evaluation in patients with narrow IACs or BCNC and (2) correlate these findings with the actual anatomical status of the cochlear nerve.

## 5. Conclusions

In CI patients, a narrow BCNC on TBCT is strongly correlated with CND and poor CI outcomes of CI. Therefore, ear, nose, and throat doctors should determine the BCNC width on preoperative CT, keeping in mind its clinical significance. Because the width of the bony cochlear nerve canal is positively correlated with long-term, post-CI speech performance, we can predict the hearing outcomes of CI by preoperatively evaluating the BCNC width.

## Figures and Tables

**Table 1:** Influencing factors on outcome of CI between 3 groups.

	Group 1	Group 2	Group 3	*p* value
	(*n* = 17)	(*n* = 14)	(*n* = 25)	
Age at CI (months)	27.92 (±15.92)	29.32 (±9.84)	33.43 (±15.7)	0.450
Hearing aids applied (months)	13.47 (±11.8)	12.47 (±9.05)	12.75 (±14.01)	0.972
PTA at CI side (dB)	94.00 (±9.98)	100.35 (±11.05)	93.40 (±9.37)	0.102
PTA at contralateral side (dB)	96.23 (±11.78)	97.92 (±12.31)	92.52 (±10.16)	0.322
BCNC width (mm)	0.91 (±0.32)	1.89 (±0.17)	2.34 (±0.14)	<0.05
CND	13/17	3/14	0/25	<0.05

CI: cochlear implantation; PTA: pure tone average; BCNC: bony cochlear nerve canal; CND: cochlear nerve deficiency. Statistical analyses by Wilcoxon rank-sum test.

**Table 2:** Results of multiple regression analysis regarding contributions of factors to the speech performance of CI.

Variable	CAP score at 24 months after CI		CAP score at 36 months after CI		Open-set word score at 24 months after CI		Picture vocabulary test (percentile) at 24 months after CI	
	*β*	*p value*	*β*	*p value*	*β*	*p value*	*β*	*p value*
Age at CI		ns		ns		ns		ns
BCNC Width		ns		ns	0.533	0.001		ns
CND		ns		ns		ns	0.346	0.015
Duration of hearing aids use		ns	−0.244	0.048		ns		ns
Residual hearing		ns		ns		ns		ns

CI: cochlear implantation; CAP: categories of auditory performance; CND: cochlear nerve deficiency; ns: nonsignificant.

**Table 3:** Technical differences and measured width of BCNC in the previous studies in normal hearing and bilateral SNHL.

	Imaging protocol	Slice chosen	Measurement	Width (mm) in control	Width (mm) in bilateral SNHL
Fatterpekar et al. [3]	Parallel to the infraorbitomeatal line	Slice at oval window	Manual measurement along the inner margin of its bony walls at its midportion	2.13 ± 0.44 (*N* = 50 ears)	1.82 ± 0.24 in profound bilateral SNHL (*N* = 33 ears)

Komatsubara et al. [10]	30° downward to the orbitomeatal line	Slice with maximum width	Digital measurement at midline between two straight lines drawn on the fundus of the IAC and the base of the modiolus	1.91 ± 0.27 (*N* = 100 ears)	0.99 ± 0.37 (with CND) 1.73 ± 0.32 (with developed cochlear nerve)

Kono [11]	Parallel to the infraorbitomeatal line	n.a	Measurement at the base of the modiolus	2.1 ± 0.2 (*N* = 118 ears)	n.a

Stjernholm et al. [2]	Temporal bone cast using silicone rubber	n.a	Measurement in axiopetrosal plane	2.58 ± 0.31 (*N* = 117 ears)	n.a
	Parallel to the infraorbitomeatal line	Slice at posterior semicircular canal	Measurement along the inner margin of its bony walls at its midportion	1.91 ± 0.24 (*N* = 100 ears)	n.a

Pagarkar et al. [12]	Direct scanning in axial plane spiral with a pitch of 1	Slice with maximum width	Maximum width was measured to the nearest 0.1 mm	1.9 ± 0.7 (*N* = 19 ears)	1.0 ± 0.2 (*N* = 8 ears)

Teissier et al. [13]	Parallel to the lateral semicircular canal	Slice containing the cochlear modiolus, the oval window	Measurement at the entry of the cochlea	2.16 ± 0.24 (*N* = 174 ears)	2.12 ± 0.55 (*N* = 120 ears)

BCNC: bony cochlear nerve canal; SNHL: sensorineural hearing loss; n.a: not available.

## References

[1] Jang J. H., Kim J. H., Yoo J. C. (2012). Implication of bony cochlear nerve canal on hearing in patients with congenital unilateral sensorineural hearing loss. *Audiology & Neuro-Otology*.

[2] Stjernholm C., Muren C. (2002). Dimensions of the cochlear nerve canal: A radioanatomic investigation. *Acta Oto-Laryngologica*.

[3] Fatterpekar G. M., Mukherji S. K., Alley J., Lin Y., Castillo M. (2000). Hypoplasia of the bony canal for the cochlear nerve in patients with congenital sensorineural hearing loss: Initial observations. *Radiology*.

[4] Miyasaka M., Nosaka S., Morimoto N., Taiji H., Masaki H. (2010). CT and MR imaging for pediatric cochlear implantation: emphasis on the relationship between the cochlear nerve canal and the cochlear nerve. *Pediatric Radiology*.

[5] Simons J. P., Mandell D. L., Arjmand E. M. (2006). Computed tomography and magnetic resonance imaging in pediatric unilateral and asymmetric sensorineural hearing loss. *Archives of Otolaryngology—Head and Neck Surgery*.

[6] Sennaroglu L. (2010). Cochlear implantation in inner ear malformations—a review article. *Cochlear Implants International*.

[7] Fatterpekar G. M., Mukherji S. K., Lin Y., Alley J. G., Stone J. A., Castillo M. (1999). Normal canals at the fundus of the internal auditory canal: CT evaluation. *Journal of Computer Assisted Tomography*.

[8] Casselman J. W., Offeciers F. E., Govaerts P. J. (1997). Aplasia and hypoplasia of the vestibulocochlear nerve: diagnosis with MR imaging. *Radiology*.

[9] Kim H.-S., Kim D.-I., Chung I.-H., Lee W.-S., Kim K.-Y. (1998). Topographical relationship of the facial and vestibulocochlear nerves in the subarachnoid space and internal auditory canal. *American Journal of Neuroradiology*.

[10] Komatsubara S., Haruta A., Nagano Y., Kodama T. (2007). Evaluation of cochlear nerve imaging in severe congenital sensorineural hearing loss. *ORL*.

[11] Kono T. (2008). Computed tomographic features of the bony canal of the cochlear nerve in pediatric patients with unilateral sensorineural hearing loss. *Radiation Medicine - Medical Imaging and Radiation Oncology*.

[12] Pagarkar W., Gunny R., Saunders D. E., Yung W., Rajput K. (2011). The bony cochlear nerve canal in children with absent or hypoplastic cochlear nerves. *International Journal of Pediatric Otorhinolaryngology*.

[13] Teissier N., Van Den Abbeele T., Sebag G., Elmaleh-Berges M. (2010). Computed tomography measurements of the normal and the pathologic cochlea in children. *Pediatric Radiology*.

[14] Nelson E. G., Hinojosa R. (2001). Aplasia of the cochlear nerve: A temporal bone study. *Otology & Neurotology*.

[15] Adunka O. F., Roush P. A., Teagle H. F. B. (2006). Internal auditory canal morphology in children with cochlear nerve deficiency. *Otology & Neurotology*.

[16] Russo E. E., Manolidis S. (2006). Cochlear nerve size evaluation in children with sensorineural hearing loss by high-resolution magnetic resonance imaging. *American Journal of Otolaryngology - Head and Neck Medicine and Surgery*.

[17] Papsin B. C. (2005). Cochlear implantation in children with anomalous cochleovestibular anatomy. *The Laryngoscope*.

[18] Adunka O. F., Jewells V., Buchman C. A. (2007). Value of computed tomography in the evaluation of children with cochlear nerve deficiency. *Otology & Neurotology*.

[19] Buchman C. A., Teagle H. F. B., Roush P. A. (2011). Cochlear implantation in children with labyrinthine anomalies and cochlear nerve deficiency: Implications for auditory brainstem implantation. *The Laryngoscope*.

[20] Zanetti D., Guida M., Barezzani M. G. (2006). Favorable outcome of cochlear implant in VIIIth nerve deficiency. *Otology & Neurotology*.

[21] Black J., Hickson L., Black B., Perry C. (2014). Prognostic indicators in paediatric cochlear implant surgery: A systematic literature review. *Cochlear Implants International*.

[22] Kang D. H., Lee M., Lee K.-Y., Lee S. H., Jang J. H. (2016). Prediction of cochlear implant outcomes in patients with prelingual deafness. *Clinical and Experimental Otorhinolaryngology*.

[23] Phan J., Houston D. M., Ruffin C., Ting J., Holt R. F. (2016). Factors affecting speech discrimination in children with cochlear implants: Evidence from early-implanted infants. *Journal of the American Academy of Audiology*.

[24] McPhee J. R., Van de Water T. R. (1986). Epithelial-mesenchymal tissue interactions guiding otic capsule formation: The role of the otocyst. *Journal of Embryology and Experimental Morphology (JEEM)*.

[25] Lefebvre P. P., Leprince P., Weber T., Rigo J.-M., Delree P., Moonen G. (1990). Neuronotrophic effect of developing otic vesicle on cochleo-vestibular neurons: evidence for nerve growth factor involvement. *Brain Research*.

[26] Sennaroglu L., Saatci I., Aralasmak A., Gursel B., Turan E. (2002). Magnetic resonance imaging versus computed tomography in pre-operative evaluation of cochlear implant candidates with congenital hearing loss. *The Journal of Laryngology & Otology*.

[27] Spoendlin H. (1975). Retrograde degeneration of the cochlear nerve. *Acta Oto-Laryngologica*.

[28] Nadol J. B., Xu W.-Z. (1992). Diameter of the cochlear nerve in deaf humans: Implications for cochlear implantation. *Annals of Otology, Rhinology & Laryngology*.

[29] Gupta S., Maheshwari S., Kirtane M., Shrivastav N. (2009). Pictorial review of MRI/CT Scan in congenital temporal bone anomalies, in patients for cochlear implant. *Indian Journal of Radiology and Imaging*.

